# Combined carbon ion radiotherapy and immunotherapy: leveraging the immunological advantages of carbon ion

**DOI:** 10.3389/fimmu.2026.1747607

**Published:** 2026-02-25

**Authors:** Ruiming Chen, Qinqin Ma, Yuan Pan, Tanglong Zhang, Zhuoya Zhang, Cuixia Di, Juntao Ran

**Affiliations:** 1 The First Clinical Medical College of Lanzhou University, Lanzhou, China; 2 Institute of Modern Physics, Chinese Academy of Sciences, Lanzhou, China; 3 Department of Radiation Oncology, the First Hospital of Lanzhou University, Lanzhou, China

**Keywords:** carbon ions radio therapy, combined therapy, heavy-ion, immunotherapy, radioimmunity

## Abstract

Immunotherapy represents a systemic approach to cancer treatment; however, its effectiveness can be limited by intrinsic tumor resistance or immunosuppressive mechanisms within the tumor microenvironment. In contrast, carbon ion radiotherapy (CIRT) demonstrates considerable immunomodulatory potential owing to its distinct biological properties. The exposure of tumor-associated antigens, increased immune cell infiltration and activity, and modifications in the immune microenvironment after CIRT provide a mechanistic rationale for combining CIRT with immunotherapy. Furthermore, strategies aimed at converting the tumor microenvironment from immunosuppressive to immunostimulatory, or those targeting immune checkpoints, are currently under active exploration. This review summarizes the latest advances in combining heavy ion radiotherapy with immunotherapy, encompassing both preclinical and clinical studies. Based on the immunological advantages of CIRT, it provides a comprehensive summary and in-depth exploration of the potential of this combined treatment modality for future clinical applications, along with a theoretical foundation to support it.

## 1 Introduction

### 1.1 Advantages of CIRT

**Figure 1:**
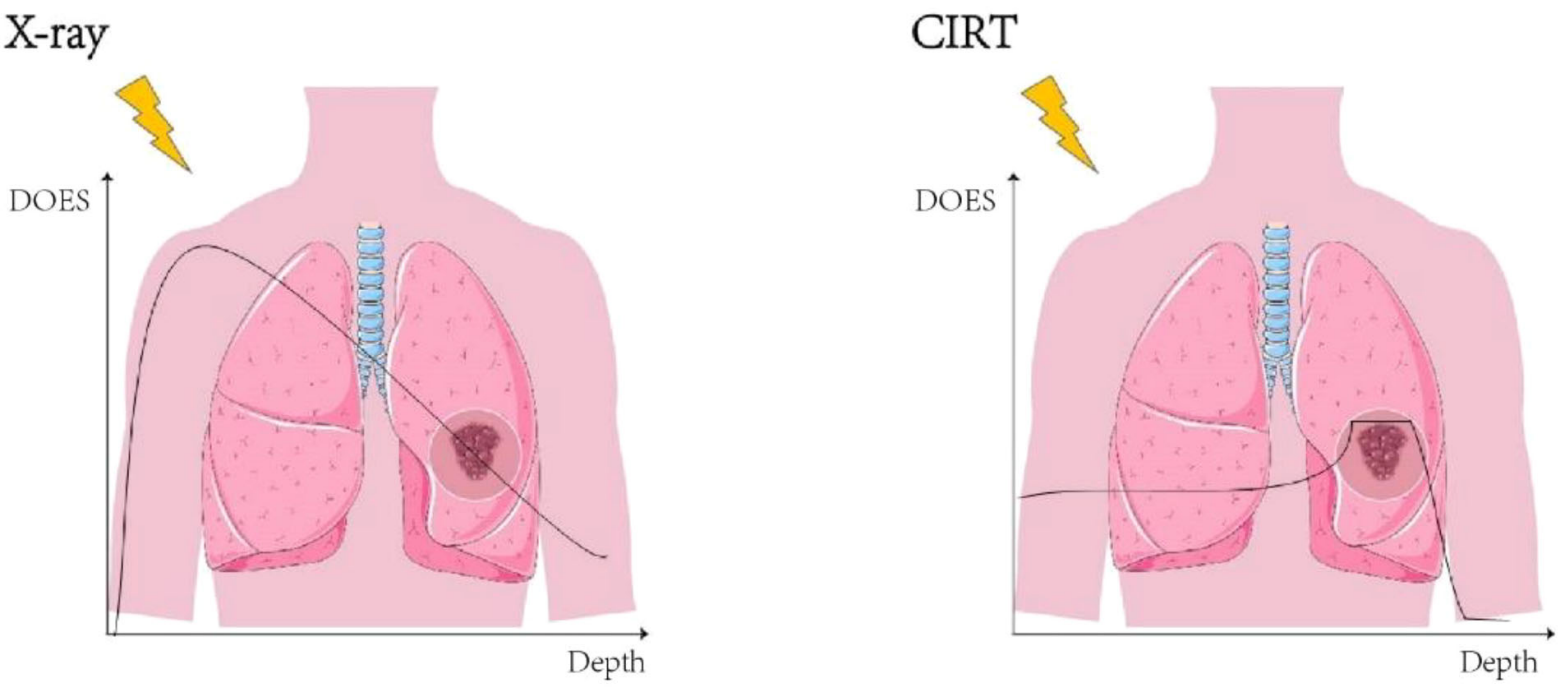
Energy deposition: heavy ions vs. X-ray. Carbon ion radiotherapy (CIRT) generate a Bragg peak within the tumor region, resulting in enhanced energy deposition precisely at the tumor site. The figure demonstrates a spread-out Bragg peak (SOBP), which provides improved coverage of the tumor volume.

Heavy-ion therapy (HIT) is currently one of the most advanced forms of radiotherapy. Heavy ions, defined as charged particles heavier than protons, encompass a range of species, including carbon, helium, neon, silicon, nitrogen, and argon, among which carbon ions are the most widely used in clinical practice (carbon ion radiotherapy, CIRT) (1). When heavy ions penetrate tissue, the velocity of the heavy ions decreases near the end of their range, leading to a sharp increase in the probability of ionization collisions and a concentrated release of energy, thereby producing the Bragg peak (2). This results in highly precise energy deposition within the tumor region. Compared with conventional X−rays therapy (XRT), this characteristic not only significantly increases the energy delivered to the tumor, but also reduces radiation damage to the surrounding normal tissues. (**Figure 1**).

Heavy ions offer a distinct radiobiological advantage over X-rays. Their high linear energy transfer (high-LET) radiation induces complex, frequently irreparable DNA double-strand breaks (DSBs) (3), which catastrophically disrupt tumor cell replication. Compared to the predominantly single-strand breaks (SSBs) generated by photon radiation, these DSBs are considerably more lethal to cells (4, 5). Current research indicates that the repair of DSBs induced by high-LET radiation proceeds at a slower rate (6). Secondly, proton radiotherapy, which also exhibits the characteristic Bragg peak, produces a sharp dose fall-off at the distal edge. However, its LET is lower than that of heavy ions, resulting predominantly in SSBs (7, 8). The DSBs caused by CIRT generate fragmented double-stranded DNA (dsDNA) that activates the *cGAS/STING–IFN* axis (9). This activation enhances tumor immunogenicity and promotes a more robust anti-tumor immune response, which may even trigger rare abscopal effects (AE). This mechanism may fundamentally explain the superior immunotherapeutic efficacy of CIRT compared to conventional XRT. Second, this distinctive property of CIRT extends to their efficacy against tumors refractory to conventional XRT (7), particularly hypoxic cells (10, 11). Moreover, CIRT exert significant cytotoxic effects even on tumors that have developed resistance after prior XRT. The characteristics of CIRT and their advantages over XRT are summarized in **Table 1**. Due to these distinct properties, CIRT is increasingly recognized as a promising and emerging modality in oncology, particularly for the treatment of tumors that have been historically refractory.

**Table 1:** CIRT versus XRT.

Characteristics		CIRT	XRT	Ref
Physical characteristics	Bragg peak	·The maximum dose is delivered by heavy ions at the termination of their trajectory, with negligible dose deposition beyond this point. ·Precisely control the Bragg peak depth within the patient. ·The SOBP can precisely cover the entire tumor volume.	The dose decreases exponentially with depth, resulting in high entrance dose, residual exit dose, and a large volume of normal tissue being irradiated.	(12)
Biological characteristics	LET	High-LET radiation	Low-LET radiation	(13)
	RBE	RBE: 2–3	RBE:1	
	Killing effect on hypoxic cells	High efficacy	Low efficacy	(10, 11)
	Types of DNA damage	less efficiently repaired, dense, spatially correlated complex clustered lesions	the majority of damages are resolved within 24 hours, discrete, simple lesions.	(4, 14, 15)
	Cell cycle dependency	Exhibits high cytotoxicity against cells in all phases of the cell cycle	Exhibits highest cytotoxicity against cells in the G2/M phase	(16)
	Immunological characteristics	·Induces stronger immunogenic cell death ·Increases the infiltration ratio of tumor immune killer cells ·Induces a stronger bystander effect ·Induces a stronger AE		(17)
Clinical application	Treatment course	Fewer fractions (4–12 sessions) and a shorter treatment course (1–3 weeks).	More fractions (20–30 sessions) and a longer treatment course (4–6 weeks).	
	Damage to tissues surrounding the tumor.	Energy is concentrated at the tumor site, and a low dose to surrounding tissues.	The energy gradually attenuates during penetration, and tissues along the path are all irradiated.	(12)
	Toxic reaction	Mini damage to normal tissues and a low incidence of grade 3+ toxicities	Prone to damaging surrounding healthy tissues	(18)

### 1.2 Limitations of immunotherapy

Immunotherapy refers to therapeutic strategies designed to activate or enhance the patient’s immune system to combat malignancies (19). Although remarkable clinical efficacy has been achieved in specific types of cancer, significant challenges remain. Treatment benefits are often limited by tumor microenvironment-mediated resistance mechanisms and other factors (20)-{{-}}-low immunogenicity and immune escape. Currently, only a very small number of patients are eligible for clinical immunotherapy, and most patients respond poorly to such treatments. Before treatment, relevant clinical examinations are required to determine whether immunotherapy can be administered. Furthermore, whether immunotherapy will yield definitive efficacy remains uncertain. Additionally, immune-related adverse events are not uncommon in clinical observations. These may include immune-mediated rash, nephritis, pneumonia, myocarditis, hypophysitis, etc., which can be life-threatening in severe cases. On the other hand, the types of tumor immunotherapy drugs currently available on the market are limited, primarily consisting of immune checkpoint inhibitors (ICIs).

### 1.3 Combination therapy — a novel treatment strategy

**Figure 2:**
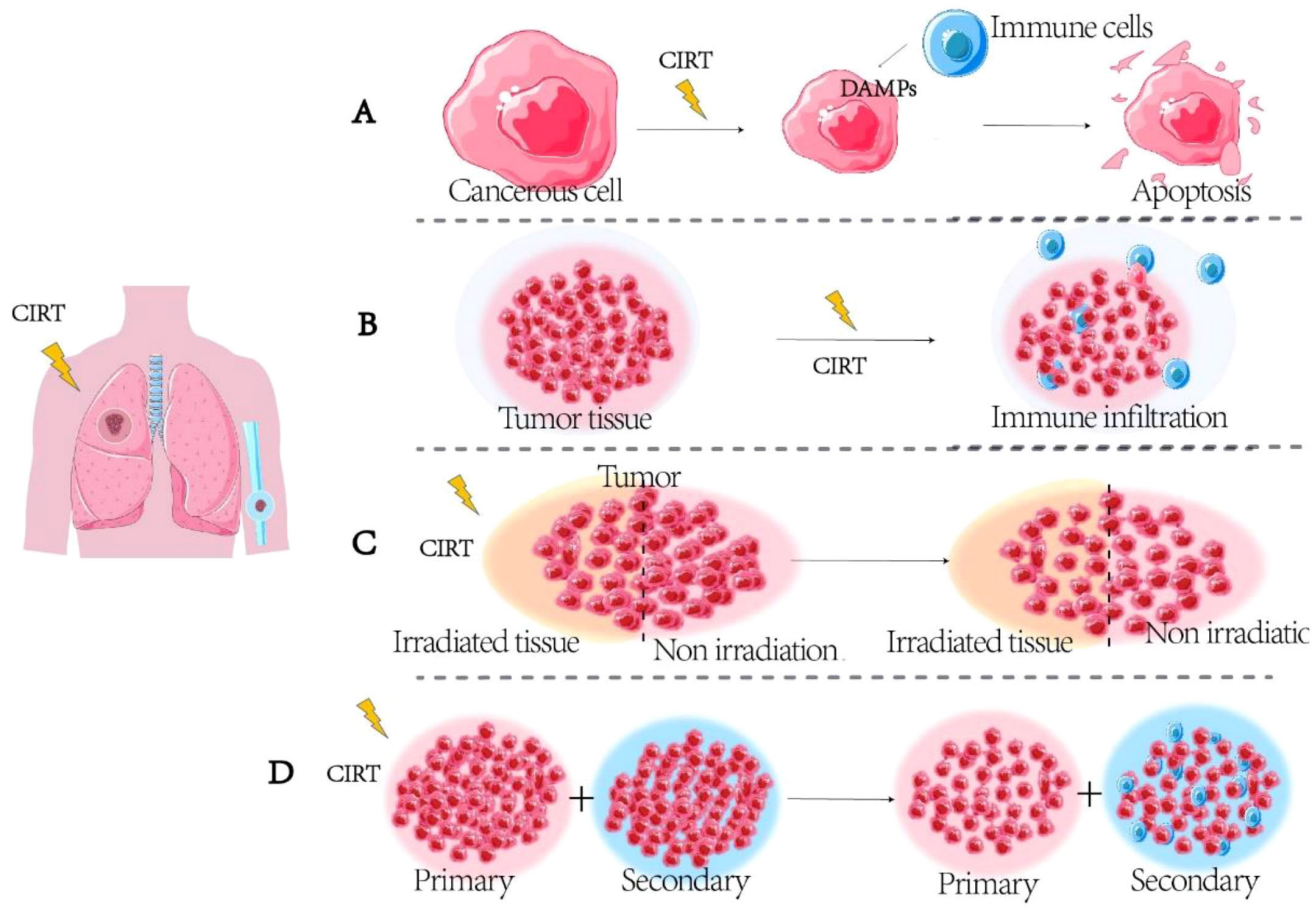
Immunological effects of CIRTA. **(A)** Induction of immunogenic cell death. **(B)** Enhanced infiltration of immune cells. **(C)** Bystander effect. **(D)** Abscopal effect (AE).

Conventional therapies exhibit limited efficacy for patients with recurrent or metastatic advanced malignancies. Currently, tumor metastasis and invasion remain among the principal causes of cancer-related mortality (21). Several studies have demonstrated that CIRT elicits a more robust immune response than conventional XRT. For example, it can increase the infiltration of tumor-infiltrating immune killer cells and induce stronger immunogenic cell death, bystander effects, and AE (**Figure 2**). This provides a feasible basis for developing combination therapies.

In a murine model of osteosarcoma metastasis, Takahashi et al. found that combining XRT (10 Gy) with immunotherapy significantly increased the distant tumor response rate compared to immunotherapy alone (complete response [CR] rate: 42% in the combination group vs. 9% in the monotherapy group). In a subsequent study (22), they further demonstrated that the combination with CIRT (5.3 Gy) also led to a significantly higher survival rate (CR rate improved from 20% with immunotherapy alone to 64% with the combination therapy) (23). Although equivalent biological doses were used, a merely literature-based comparison cannot provide an objective and comprehensive assessment of the immunological differences between CIRT and XRT, such as disparities in survival outcomes. Helm et al. established an osteosarcoma metastasis model using identical doses(10 Gy) of CIRT and XRT. They found that, for both radiotherapy modalities, combination with immunotherapy resulted in superior therapeutic efficacy compared to radiotherapy alone. While no significant difference was observed between CIRT and XRT in controlling distant tumors or in the metastasis model, the combination of CIRT with immunotherapy was strikingly superior to XRT combined with immunotherapy in controlling pulmonary metastases (6). A growing body of research substantiates the immunological advantages of CIRT over XRT. Yet, no study to date has undertaken a comprehensive comparative analysis of the two modalities.

Radiotherapy combined with immunotherapy (24) has emerged as a promising strategy for managing metastatic disease. For instance, the combination of radiotherapy with pembrolizumab has been clinically implemented in the treatment of non-small cell lung cancer (NSCLC). This combined modality demonstrates superior therapeutic efficacy and prolonged overall survival in patients with advanced NSCLC compared to monotherapy approaches (25). Therefore, by leveraging the radiobiological characteristics of heavy ions and considering them as immunomodulators, we can combine them with immunotherapy to potentiate anti-tumor immune responses. The AE represents a potential therapeutic opportunity for metastatic cancers and is widely attributed to radiation-induced immunomodulation (26). Compared to conventional radiotherapy, CIRT demonstrates greater biological effectiveness and enhances post-irradiation tumor immunogenicity (27), thereby providing a mechanistic basis for investigating the systemic anti-tumor immune response induced by heavy ions. With advancing research on CIRT induced immunomodulation, the combination of radiotherapy and immunotherapy has emerged as a promising therapeutic strategy for advanced-stage malignancies (28).

Research into radiation-induced remodeling of the tumor immune microenvironment is now expanding rapidly. These immunological insights are crucial for elevating radiotherapy from a local intervention to a systemic treatment. This review summarizes key developments in combining CIRT with immunotherapy, with a focus on promising clinical strategies. It also evaluates the present state of both preclinical and clinical research in this field.

## 2 CIRT enhance anti-tumor immunity

### 2.1 Exposure of antigens via the cGAS/STING pathway by CIRT

**Figure 3:**
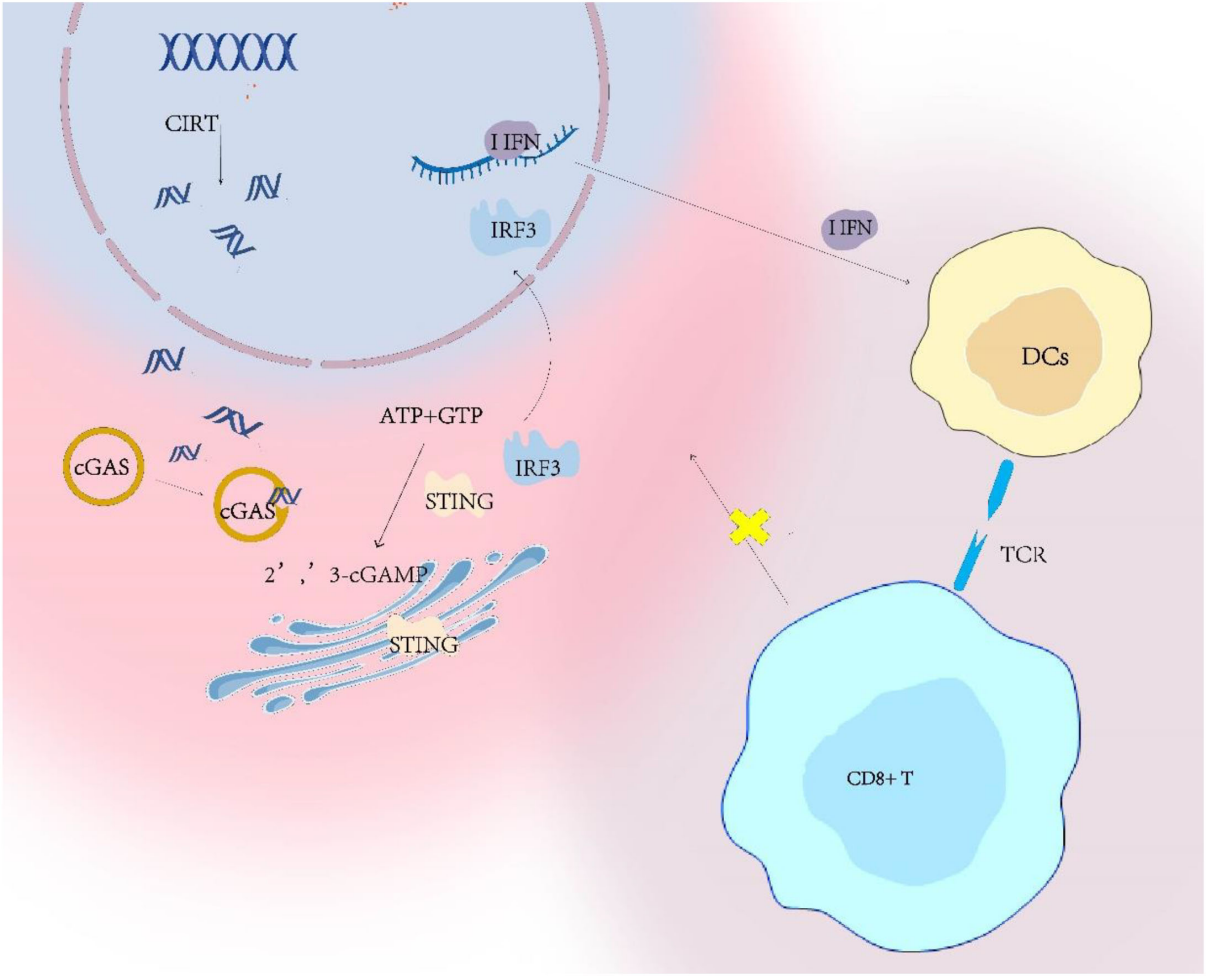
The dsDNA-cGAS/STING-I IFN axis activates DCs and enhances the tumor-killing activity of CD8^+^ T cells.

CIRT alters both the tumor microenvironment and subcellular structures, thereby enhancing the exposure of tumor antigens. As discussed previously, CIRT leads to increased DSBs. The cytosolic dsDNA is recognized by cyclic GMP-AMP synthase *(cGAS*) *(*29), which catalyzes the synthesis of the cyclic dinucleotide 2′3′-cyclic guanosine monophosphate–adenosine monophosphate *(cGAMP)*. *cGAMP* activates the stimulator of interferon genes (*STING*), ultimately resulting in *STING* phosphorylation (30) (**Figure 3**). One of its hallmark features being the induction of type I interferon *(IFN)* secretion (31). *IFN* have been shown to inhibit tumor cell proliferation and disrupt tumor vasculature (32). Furthermore, studies indicate that *STING* signaling can also be associated with the activation of transcription factors such as nuclear factor-kappa B (*NF-κB*) (33). Hellweg et al. investigated the relationship between *NF-κB* and high-LET radiation, further demonstrating that both *ATM* inhibitors and proteasome inhibitors can suppress this activation. These results underscore the critical roles of *ATM* and the proteasome in mediating CIRT induced *NF-κB* signaling (34). The potential of *STING* signaling in cancer immunotherapy stems from its ability to promote diverse anti-tumor immune responses.

In addition, following CIRT, the activation of apoptotic genes and the release of immunogenic tumor-associated antigens work together to promote the death of tumor cells. A meta-analysis showed that exposure to high-LET radiation triggered substantial transcriptional changes within 6–24 hours, resulting in the upregulation of 2,689 genes and the downregulation of 1,839 genes. The most notable differential expression pattern featured the upregulation of autophagy-related genes alongside the downregulation of DNA repair-associated genes (35). Compared to conventional XRT, CIRT induces not only *p53*-mediated gene upregulation but also more substantial and more prolonged transcriptional activation at 24 hours post-irradiation (36). Furthermore, Sofia et al. showed that high-LET radiation upregulates a broad spectrum of immunologically relevant molecules at the cellular level, including immune recognition molecules *(HLA, ICAM-1, CEA, MUC-1)*, surface-exposed calreticulin (CRT), damage-associated molecular patterns *(HMGB1, ATP)*, and the immune checkpoint protein *PD-L1 (*37). A growing body of experimental evidence confirms that CIRT induce similar molecular and cellular alterations, which represent promising therapeutic targets for enhancing radiation-induced tumor immunogenicity. These findings collectively indicate that tumor cells acquire an enhanced immunogenic potential following CIRT.

### 2.2 Enhancement of immune response through TME remodeling by CIRT

**Figure 4:**
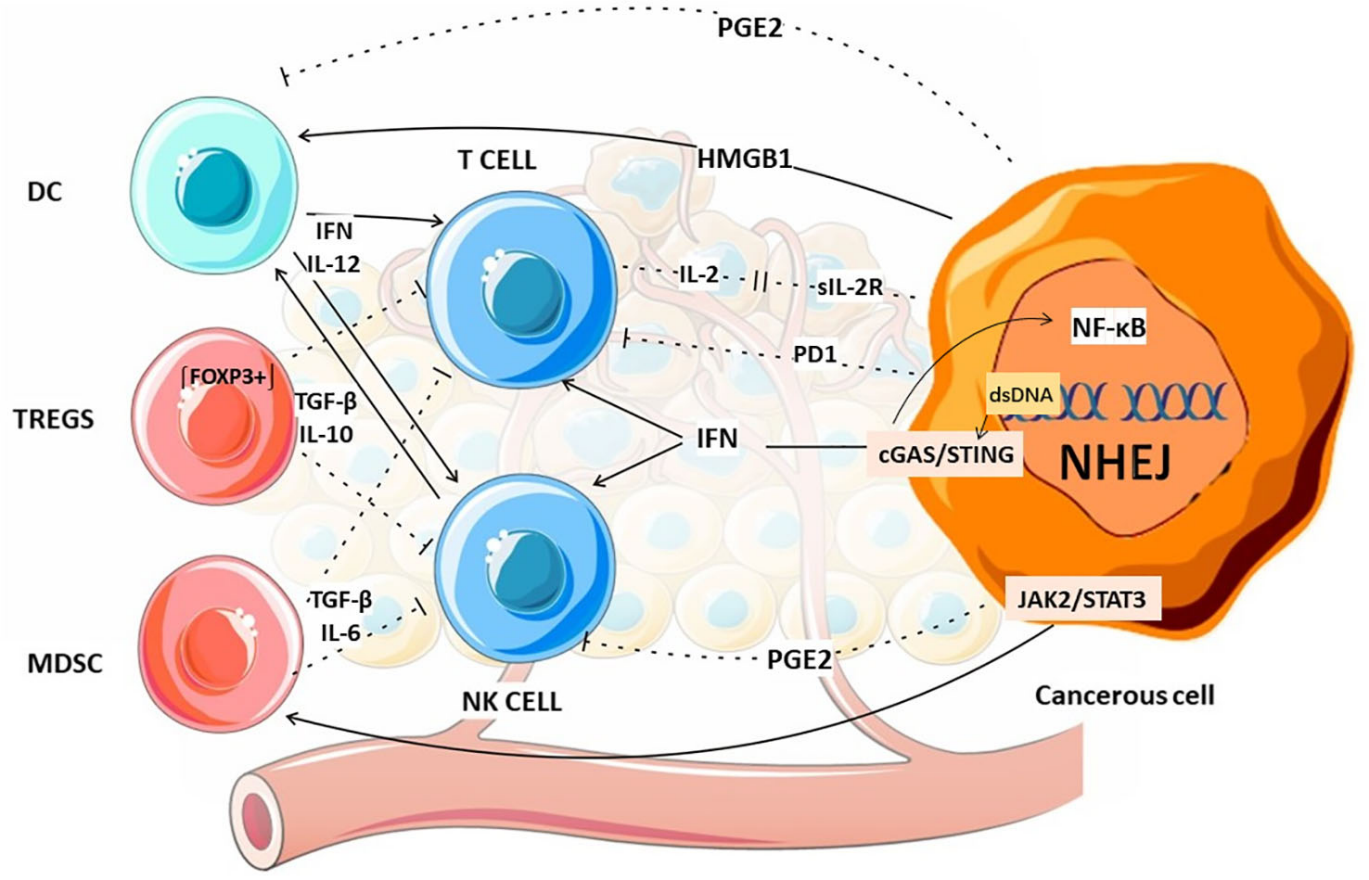
The expression changes of some substances in cells after CIRT.

Investigating changes in the tumor microenvironment (TME) after CIRT and combining this modality with immunotherapy to promote an anti-tumor immune milieu constitutes a core research paradigm. Enhancing the immune system’s ability to recognize and eliminate tumor cells is a central objective in tumor immunology. Sofia et al. further investigated radiation-induced alterations in the tumor immune microenvironment and revealed a series of coordinated changes at the macro level: increased infiltration of cytotoxic T lymphocytes (CD8^+^) and helper T cells (CD4^+^), enhanced natural killer (NK) cell activity, maturation of DCs, decreased populations of regulatory T cells (Tregs), and suppressed function of myeloid-derived suppressor cells (MDSCs) (37). These alterations collectively demonstrate a significant enhancement of anti-tumor immunity following irradiation. The established mechanism of immune-mediated tumor control operates precisely through these pathways (**Figure 4**).

Experimental evidence from post-irradiation immunological profiling suggests that CIRT have a significant immunomodulatory effect on host immune competence. For instance, localized CIRT delivery to cerebral targets has been shown to recruit non-targeted microglia to the treatment volume and enhance their immunophagocytic capacity (38). A recent study revealed that the epidermal growth factor receptor ligand amphiregulin *(AREG)* is associated with distant metastasis following radiotherapy (39). This phenomenon was observed in both murine and human models. Using a mouse model, the researchers further demonstrated that *anti-AREG* therapy could inhibit radiation-induced metastatic tumor growth and enhance the efficacy of radiotherapy. This finding provides a feasible mechanistic rationale for suppressing metastatic proliferation after radiotherapy. Patients with metastatic cancer may thus benefit from combined radiotherapy and immunotherapy. Takeshima et al. compared the efficacy of immunotherapy combined with either CIRT or XRT in mice (40). They made a critical observation: the attenuation of tumor volume reduction after CD8^+^ T cell depletion was far more pronounced in the heavy-ion group, implying that CIRT relies more extensively on CD8^+^ T cell-mediated immunity. This finding suggests that heavy-ion radiation may have superior compatibility with immunotherapy and the potential for enhanced therapeutic outcomes. Further exploring this synergy, Huang et al. examined the combined effect of CIRT and ICIs in a murine tumor model (41). The study demonstrated that the combined treatment significantly inhibited both local and distal tumor growth and prolonged survival in mice. Transcriptomic sequencing and bioinformatic analyses revealed a significant enrichment of immune-related pathways and genes in the combination group, accompanied by enhanced infiltration of immune cells and increased secretion of cytokines and chemokines. These findings provide experimental evidence supporting the combined use of CIRT and ICIs in cancer therapy. Hartmann et al. established a bilateral tumor model using Her2+ EO771 murine breast cancer cells. Their findings demonstrated that the combination of CIRT with anti-*CTLA-4* therapy yielded superior antitumor efficacy compared to either monotherapy and induced AE. Notably, when mice that had undergone combined radioimmunotherapy were rechallenged with tumor cells, they exhibited complete resistance to tumor establishment. This observation suggests that the combined treatment can induce durable, tumor-specific immune memory (42). While such findings collectively demonstrate the potential of combination therapy, numerous challenges remain in its translation to clinical application. Taken together, these studies indicate that CIRT exerts synergistic effects with immunotherapy through enhanced immune reliance, activation of the tumor immune microenvironment, and the induction of immune memory. This provides a strong experimental basis for developing combined treatment strategies in the clinic.

Radiation-induced lymphopenia (RIL) is a known side effect of radiotherapy (43). When XRT with large radiation fields encompasses tumor-draining lymph nodes as well as hematopoietic and lymphoid organs such as the bone marrow, spleen, and thymus, both the lymphatic circulation and hematopoiesis can be compromised, leading to a reduction in lymphocytes. Lymphocytes are essential for mounting effective anti−tumor immune responses. In contrast, proton and heavy ions beams exhibit a sharp dose fall−off at the field edges, resulting in less damage to surrounding lymphoid tissues and thereby preserving a greater number of functional lymphocytes. A recent statistical analysis further demonstrated that patients treated with X-rays exhibit consistently lower relative lymphocyte counts upon treatment completion than those receiving ion therapy. This finding solidifies the aforementioned perspective (44).

## 3 Utilizing immunotherapy for radiosensitization

### 3.1 Combination of target inhibitors/agonists

#### 3.1.1 Combination with STING agonists

*STING* agonists enhance antitumor immune responses by activating the *cGAS*-*STING* pathway and promoting the secretion of *IFN* and pro-inflammatory cytokines. Although these drugs are currently being evaluated in Phase I/II clinical trials, including in combination with immunotherapies (e.g., pembrolizumab) (45), no *in vivo* studies have been conducted on their combination with CIRT. Nevertheless, existing theoretical research suggests this combination represents a feasible therapeutic strategy.

#### 3.1.2 Combination with NHEJ inhibitors

Since CIRT primarily induce DSBs, their tumoricidal effect can be potentiated by strategies that enhance damage or impair repair, such as the inhibition of non-homologous end joining (NHEJ). Ma et al. tested this by combining CIRT with the DNA repair inhibitors NU7026 (NHEJ inhibitor) and B02 (HR inhibitor). The study revealed a marked, concentration-dependent enhancement of tumor sensitivity to both CIRT and XRT with NU7026, in contrast to only a modest effect from B02 (46). High-LET radiation inherently suppresses NHEJ repair, and the specific inhibition of this pathway further compromises it. Such dual interference substantially enhances the sensitivity of cancer cells to CIRT. Furthermore, these results also demonstrate that CIRT primarily induces DNA damage that is repaired via NHEJ. In an experimental study using human lung adenocarcinoma cells (A549), Yang and colleagues not only confirmed these findings but also demonstrated that NU7026 pretreatment reduced the post-irradiation survival fraction to 0.67% following CIRT —significantly lower than that observed with XRT(8.75%). The survival fractions in control groups were as follows: untreated cells, 98.75%; NU7026 alone, 90.82%; and the *ATM/ATR* inhibitor CGK733 alone, 92.63%. Flow cytometry analysis revealed that NU7026-pretreated A549 cells exhibited a significantly increased G2/M arrest at 24 hours post-CIRT (*P < 0.05 and P < 0.001*). This arrest was diminished by 48 hours as the apoptotic population increased substantially. Further experimental analysis confirmed a marked upregulation of both *ATM and ATR* at both the gene and protein levels in NU7026-pretreated cells after CIRT (47).

Building on the established biological mechanisms of NHEJ inhibitors, clinical trials are currently exploring their therapeutic potential in combination with chemotherapy or XRT. Although no published studies have yet reported their use with CIRT, compelling preclinical evidence suggests that combining NHEJ inhibition with CIRT represents a transformative strategy for future oncological applications. Nedisertib can block NHEJ repair (48). Currently, research on Nedisertib has been conducted in head and neck cancer (49), glioblastoma (50), acute myeloid leukemia (51), and colorectal cancer (52). Current research on NHEJ inhibitor-radiotherapy combinations remains confined to *in vitro* studies (53), with no clinical trial results reported to date. Nevertheless, these findings robustly demonstrate that NHEJ serves as the primary DNA repair pathway following exposure to high-LET radiation. Moreover, they suggest that combining CIRT with NHEJ inhibitors may represent a novel therapeutic paradigm, offering a promising strategy to enhance radio-sensitivity in CIRT.

#### 3.1.3 Combination with NF-κB inhibitors

The activation of *NF-κB* promotes tumor immune escape by inhibiting apoptosis and enhancing proliferation in tumor cells. Current evidence indicates that CIRT activates the *NF-κB* pathway through the induction of DSBs (54), by the *cGAS*/*STING* axis. ATM inhibitors can suppress the repair of DSBs. Further studies on the radiosensitizing effects of *ATM* inhibitors have shown that, although the degree of sensitization varies among cell lines, all tested groups exhibited significantly enhanced radiation sensitivity compared to controls (55). Given that CIRT induce substantial DSBs, subsequent research has addressed this variability by demonstrating that the inhibitor’s radiosensitizing effect is significantly more pronounced when combined with high-LET radiation (56).

These findings offer critical insights into enhancing radiation-induced tumor cell death by modulating the *NF-κB* pathway. Inhibiting key intermediates in this signaling cascade can effectively counteract tumor escape mechanisms after radiotherapy. Thus, *NF-κB* represents a promising therapeutic target in oncology, and its strategic combination with radiotherapy holds strong theoretical potential for improving treatment outcomes. It is noteworthy that current research on *NF-κB* pathway modulation has also been applied to the radioprotection of normal tissue after radiotherapy. However, similar to the development of NHEJ inhibitors, studies on combining *NF-κB*-targeted therapy with radiotherapy remain confined mainly to preclinical stages, with clinical translation still lacking.

### 3.2 Combination therapy reprograms the TME

#### 3.2.1 Amplify T cell cytotoxic efficacy against tumors

The increased infiltration of CD8^+^ T cells into the tumor microenvironment is a critical event in antitumor immunity. A primary research focus is therefore directed toward developing strategies that enhance tumor-localized recruitment and accumulation of these effector cells. Given its immunostimulatory properties, CIRT represents a promising modality for achieving this goal, making it a central focus in radio-immunotherapy research.

##### 3.2.1.1 Combination of CIRT with ICIs

*PD-1* inhibitors are a class of therapeutic agents that bind specifically to *PD-1*, blocking the *PD-1*/*PD-L1* signaling pathway and thereby restoring T cell cytotoxicity (57). In contrast to the previously described concept that CIRT promote anti-tumor immunity, CIRT has been shown to notably upregulate *PD-L1* expression. The interaction between *PD-1* and *PD-L1* serves as a crucial immune checkpoint(ICIs), suppressing the function of immune cells, particularly CD8^+^ T cells. For instance, Tiar et al. reported that CIRT induced higher PD-L1 expression in human osteosarcoma U2OS cells than XRT (58). In murine models, Zhou et al. further demonstrated that combining CIRT with *PD-1* immune checkpoint blockade significantly increased the proportions of CD4^+^ and CD8^+^ T lymphocytes in both splenic and tumor tissues (59). In summary, although radiotherapy promotes *PD-L1* upregulation—a potential adaptive immune resistance mechanism—combining radiotherapy with *PD-1* inhibitors yields superior antitumor efficacy compared to either treatment alone (60).

Notably, unlike other immune-targeted therapies that remain confined to preclinical studies, *PD-1* inhibitor-based radioimmunotherapy has already been successfully translated into clinical applications. Currently, *PD-1* inhibitors (e.g., nivolumab and pembrolizumab) have been clinically approved for the treatment of various malignancies, including melanoma, NSCLC, and esophageal squamous cell carcinoma. The combination therapy of radiation and *PD-1* inhibitors has successfully transitioned from bench to bedside, demonstrating the successful translation of basic research into clinical applications. Research on the combination of radiotherapy and ICIs continues to advance beyond these findings. Recent preclinical studies have shown that combining *STING* agonists with *PD-L1* inhibitors results in significantly enhanced antitumor efficacy. This synergistic approach reduces *PD-L1* expression in tumor-associated monocytes while amplifying *STING* pathway activation, thereby promoting T cell infiltration and activation for T-cell infiltration and activation to facilitate a more robust antitumor immune response (61). This study highlights the tremendous potential of dual immunotherapy combinations while also illuminating promising future directions for combining radiotherapy with multiple immunomodulatory agents—including novel radiation modalities. As T lymphocytes serve as pivotal effectors in antitumor immunity, investigating CIRT modulation of tumor-infiltrating T cells represents a critical research axis for advancing combined radioimmunotherapy strategies. However, pivotal questions emerge: despite the reduced damage to lymphocytes in the tumor region compared to XRT, does CIRT still lead to a significant decrease in the absolute number of local immune cells? Furthermore, if RIL occurs, would the subsequent combination with ICIs achieve synergistic therapeutic efficacy, or merely lead to an additive burden of adverse effects? And is this dose-dependent?

##### 3.2.1.2 Combination of CIRT with either IL-2 agonists or sIL-2R inhibitors

An et al. demonstrated in a hamster cheek pouch carcinoma model that CIRT induced a dose-dependent reduction in serum soluble interleukin-2 receptor (*sIL-2R*) levels (62), revealing an inverse correlation between radiation dose and *sIL-2R* concentration. *IL-2*, primarily secreted by activated CD4^+^ T cells, plays a pivotal role in promoting anti-tumor immunity (63). Tumor cells exploit *sIL-2R* overexpression as an immune evasion mechanism, in which *sIL-2R* competitively binds *IL-2* and impedes its interaction with membrane-bound *IL-2* receptors *(mIL-2R*), thereby suppressing IL–2–mediated T cell activation (64). These findings suggest that combining CIRT with *IL-2*–stimulating agents or *sIL-2R*–depleting therapies may enhance antitumor immunity by promoting T cell infiltration. However, the underlying mechanisms require further experimental validation.

##### 3.2.1.3 Combination of CIRT with CAR-T

Chimeric antigen receptor T (CAR-T) therapy, as an emerging oncologic treatment modality (65), demonstrates substantial potential when combined with CIRT. Wang et al. developed a B7-H3-targeted CAR-T cell therapy and found that, when combined with radiotherapy, it demonstrated superior efficacy in eliminating radioresistant chordoma cells compared to either CAR-T cell therapy or radiotherapy alone (66). Mechanistic studies suggest that radiation enhances tumor antigen exposure and remodels the immunosuppressive microenvironment, thereby potentially improving CAR-T cell infiltration and sustained activity. However, there is currently no definitive research confirming that CIRT also holds significant superiority. Theoretically, it is postulated that CIRT can induce more potent immunogenic cell death and release a greater amount of tumor-associated antigens, which could provide more targets for CAR-T therapy and enhance the antigen recognition and activation of CAR-T cells.

#### 3.2.2 Amplify NK cell cytotoxic efficacy against tumors

NK cells are also regarded as important tumor killer cells. Studies demonstrate that CIRT may upregulate *NKG2D-L* expression on tumor cells (67), thereby enhancing NK cell infiltration into tumor tissue (68). NK cells exhibit intrinsic tumoricidal activity that operates independently of prior sensitization and MHC restriction, demonstrating their distinct advantages in antitumor immunity.

A novel perspective has been proposed: NK cells can also modulate tumor immunity through their regulatory effects on other immune cell populations (69). The study revealed that NK cells orchestrate a comprehensive antitumor immune response by recruiting conventional type 1 dendritic cells (cDCs) to the tumor microenvironment through chemokine-mediated mechanisms, subsequently initiating a T-cell activation cascade. CIRT upregulates *NKG2D* expression on tumor cells, which augments NK cell-mediated cytotoxicity (70). Targeting *NKG2D* with CAR-NK cells could leverage this mechanism to enhance combination therapy (71). In summary, recent research has predominantly focused on CD8^+^ T cells and NK cells when investigating radiation-induced immunological changes in the tumor microenvironment, given their pivotal roles in mediating tumor cytotoxicity.

#### 3.2.3 Attenuate the immunosuppressive activity of Tregs

A recent study revealed a significant reduction in tumor-infiltrating FOXP3^+^ Tregs in cervical cancer patients following radiation therapy (72). Tregs mediate immunosuppression by secreting inhibitory cytokines such as TGF-β and IL-10 (73), which impair the function of effector immune cells including T cells and NK cells, thereby compromising antitumor immunity. Furthermore, Tregs promote tumor angiogenesis by upregulating pro-angiogenic factors, particularly vascular endothelial growth factor (VEGF), thereby facilitating tumor vascularization and progression (74). A meta-analysis indicated that elevated FOXP3^+^ expression is significantly associated with reduced overall survival in multiple malignancies such as NSCLC and cervical carcinoma. However, certain tumor types showed an inverse correlation (75). Therapeutic depletion of Tregs using anti-CD25 monoclonal antibodies (e.g., daclizumab) (76) or immunotoxins (e.g., denileukin diftitox) (77) enhances antitumor immune responses. However, a clinical trial of denileukin diftitox in advanced breast cancer achieved disease stabilization in only a minority of patients (4 patients; 27%, 95% CI [0.08, 0.55]) (78). Future studies should investigate alternative dosing regimens that yield more consistent Treg depletion and enhanced safety profiles. Additional research is also warranted to evaluate whether combining CIRT with Treg inhibitors represents a more feasible alternative with reduced toxicity.

#### 3.2.4 Attenuate the immunosuppressive activity of MDSCs

CIRT reduces MDSCs populations through a *JAK2/STAT3* dependent mechanism (79). MDSCs exert comprehensive immunosuppressive effects through multiple interconnected mechanisms, including T cell functional impairment mediated by arginine depletion (80) and NO production (81), secretion of inhibitory cytokines such as *TGF-β (*82) and *IL-6* (83) that suppress antitumor immunity, and the generation of secondary immunosuppressive cell populations that further contribute to immune evasion (84). The mechanistic link between MDSCs functional inhibition and enhanced immune-mediated tumor control establishes the molecular basis for the superior immunotherapeutic efficacy of CIRT compared to conventional radiotherapy. The immunomodulatory effects of CIRT are particularly evident in the regulation of MDSCs, suggesting that combining CIRT with pharmacological agents targeting MDSCs suppression may potentiate tumor radiosensitivity.

#### 3.2.5 Amplify DCs functionality

CIRT induces *HMGB1* release from human cancer cells (85). Onishi et al. demonstrated that radiation-induced *HMGB1* release exhibits LET-dependence, with higher LET radiation more effectively promoting *HMGB1* release and subsequent antitumor immunity (86). *HMGB1* potentiates DCs activation, ultimately eliciting antitumor immune responses (87). CIRT significantly enhances interferon-gamma (IFN-γ) and interleukin-12 (IL-12) secretion by mature DCs (88). Tumor cells irradiated with CIRT demonstrate the capacity to improve DCs expression of CD40 and IL-12. These activated DCs exhibit significant anti-metastatic activity *in vivo (*89).

## 4 Application

### 4.1 Strategies to increase AE rates

Investigating the AE represents the key to evolving radiotherapy from a localized treatment into a systemic therapy. Brenneman et al. reported a patient with metastatic retroperitoneal sarcoma who exhibited AE following proton therapy (90). Through comprehensive immunohistochemical analysis of both primary and metastatic lesions, they identified a *SMARCB1* nonsense mutation along with loss of *INI1* protein expression, suggesting these molecular changes may be linked to the observed AE. However, no in-depth mechanistic investigations were conducted to clarify the underlying biological pathways. To decipher the mechanisms of AE, studies examining the immunohistochemical features of primary and metastatic tumors, alterations in the TME, and systemic immune changes following its occurrence are critically needed.

### 4.2 Clinical application implications

CIRT has demonstrated promising clinical outcomes in some tumor types, achieving excellent local control rates, survival benefits, and manageable toxicity profiles (91, 92). However, there remains a paucity of research investigating the immunomodulatory effects of CIRT or its combinatorial potential with immunotherapy. In addition to investigating the potential of combination therapies, research into their dosage, sequencing, and adverse effects is equally critical. Hu et al. compared peripheral blood samples from prostate cancer patients before and after treatment with either CIRT or XRT. Their analysis confirmed that, unlike XRT, CIRT did not induce significant lymphocytopenia. Notably, most lymphocyte subsets (except Tregs) exhibited enhanced proliferative capacity following particle therapy, demonstrating an immunological advantage over XRT. Additionally, elevated *TNF-α* levels were detected post-treatment, which may promote NK cell proliferation and thereby enhance NK cell-mediated antitumor immunity (93). Although relevant studies have provided some basis for understanding RIL, the current knowledge remains limited and contains gaps. Ronchi et al. treated 40 patients with head and neck mucosal melanoma using CIRT, among whom 18 received immunotherapy either before or after irradiation. Statistical analysis revealed a significantly prolonged median survival time of 17 months in the combination therapy group compared to 3.6 months in the CIRT-alone group *(p < 0.001*) (91). Mizoguchi et al. analyzed 34 patients with newly diagnosed head and neck mucosal melanoma treated with primary CIRT (total dose 64 Gy). The patients were divided into three groups: Group A (radiotherapy alone), Group B (radiotherapy followed by ICIs therapy with Nivolumab), and Group C (concurrent radiotherapy and ICIs therapy). Post-treatment follow-up revealed a two-year overall survival rate of 100% in Group C (11/11), compared to 66.7% in Group B and 50.8% in Group A. Differential analysis confirmed a statistically significant difference between Group C and Group A, whereas the difference between Group B and Group A was not statistically significant (94). Undoubtedly, this study provides clinical evidence to inform the timing of combination therapy, thereby addressing one of its key challenges. However, the small sample size limits the generalizability of the findings. Given that head and neck mucosal melanoma is a rare tumor, applying similar research approaches to diseases with higher incidence would yield more clinically actionable guidance. Clinical trials investigating the combination of CIRT with immunotherapy are being conducted actively at major particle therapy centers. At present, most of the studies on CIRT combined immunotherapy are retrospective studies, or immunotherapy that is received before and after CIRT, without concurrent treatment. Therefore, prospective clinical studies on CIRT combined immunotherapy are being actively carried out (**Table 2**). These also indicate that the primary reason for the clinical trial**’**s failure was an insufficient number of enrolled patients.

**Table 2:** Clinical research on CIRT combined with immunotherapy.

Registration number	Country	Clinical trials	Drug	Tumor type	Status	Reason for stopping	Ref
NCT02946138	China	Phase II Trial	GM-CSF	Hepatocellular Carcinoma	Withdrawn	Enrollment was too slow	(95)
NCT05229614	Germany	Phase II Trial	Pembrolizumab	Solid cancer	Recruiting		(96)
NCT06805864	Japan	Phase II Trial	Pembrolizumab	Cervical Adenocarcinoma	Terminated	Not yet recruiting	(97)
NCT04143984	China	Phase II Trial	Camrelizumab	Nasopharyngeal Carcinoma	Recruiting		(95)

### 4.3 Toxicity profiling and intervention

After combination therapy, research on treatment-related toxicities has emerged as another critical focus area. A meta-analysis of animal studies comparing immunotherapeutic agents (ipilimumab and *IL-2*) as radiosensitizers versus conventional radiosensitizers (cisplatin) demonstrated that immunotherapy significantly enhances local tumor control when combined with radiotherapy, without exacerbating radiation-induced toxicity (98). There are data that suggest that CIRT-immunotherapy synergy may confer a significant survival advantage over CIRT alone (41). Cavalieri et al. conducted a retrospective analysis of adverse events in 33 patients treated with combined CIRT and immunotherapy, revealing no significant increase in toxicity incidence (99). Currently, there remains a paucity of relevant studies. However, existing evidence has not identified significantly additive toxic effects when combining immunotherapy with radiotherapy. Should future large-scale studies conclusively demonstrate that combination therapy does not exhibit substantially greater toxicity than either modality alone, this would provide compelling evidence to support broader clinical adoption of combined treatment approaches.

The studies mentioned above are based on investigations of radiation toxicity. Whether the combination of CIRT and immunotherapy would exacerbate immune-related adverse events remains unexplored. A retrospective study systematically evaluating the safety and toxicity of stereotactic body radiotherapy (SBRT) combined with ICIs for lung cancer revealed that the incidence of grade 3 or higher pneumonitis in the SBRT + ICIs group was 10.7%, significantly higher than that in the SBRT monotherapy group (*0%; P = .007)*. However, the incidence of pneumonitis of any grade showed no statistically significant difference between the two groups (100). Unfortunately, the study did not report the median overall survival or tumor control rates for either group, leaving it unclear whether the observed pneumonitis was associated with antitumor efficacy.

### 4.4 Next-generation therapeutic blueprints and their clinical translation trajectories

#### 4.4.1 Innovations in radiotherapy technology

Beyond conventional CIRT- ICIs combinations, emerging radiation techniques including FLASH radiotherapy and microbeam radiation therapy are currently under active investigation. Bertho et al. conducted a comparative study evaluating the therapeutic efficacy of carbon ion microbeam therapy versus conventional CIRT in a murine osteosarcoma model. Microbeam radiation therapy is an emerging treatment modality that employs spatially fractionated beams divided into submillimeter-width microbeams. These microbeams create alternating high-dose regions (peak doses) and low-dose regions (valley doses) in tissue, typically spaced several hundred micrometers apart. This approach significantly reduces normal tissue toxicity while simultaneously enhancing immune responses. Comparative studies indicate that while conventional broad-beam CIRT induces greater CD8^+^ T-cell infiltration, microbeam radiation therapy achieves comparable tumor coverage (over 70% of the tumor volume) with a substantially lower absorbed dose of only 1.5 Gy (101). Meanwhile, carbon-ion FLASH radiotherapy may enhance antitumor immunity by downregulating *PD-L1* expression while upregulating calreticulin (*CRT*) and MHC class I presentation, thereby promoting immunogenic cell death and improving T-cell recognition (102). The integration of these emerging CIRT techniques with ICIs represents a promising new paradigm in radiation oncology research.

#### 4.4.2 Innovations in immunotherapy approaches

When combined with ICIs, this strategy significantly enhances antitumor immunity (**Table 3**). Jiang et al. developed *KN046@19F-ZIF-8*, a novel dual-targeting agent that simultaneously blocks both *PD-L1* and *CTLA-4*. *In vivo* studies demonstrated a substantially increased proportion of T-cells in the tumors and spleens of mice treated with this formulation compared to controls. Additionally, the KN046@19F-ZIF-8 group showed a marked reduction in Treg populations (110). These findings align with the known radiobiological properties of CIRT, suggesting that combining this dual-targeting agent with CIRT could represent a highly promising therapeutic strategy.

**Table 3:** Partial immunotherapy methods.

Category	Representing	Mechanism	Application	Ref
Immune checkpoint inhibitors(ICIs)	Pembrolizumab Nivolumab	PD-1 blockade	NSCLC Advanced melanoma	(103)
	Atezolizumab Durvalumab	PD-L1 blockade	Classical Hodgkin’s lymphoma	(104)
	Ipilimumab	CTLA-4 blockade	NSCLC	(105)
	Relatlimab	LAG-3 inhibition blockade	Melanoma (combination with nivolumab)	(106)
Bispecific checkpoint inhibitor	Cadonilimab	PD-1/CTLA-4 bi-specific antibody	Cervical cancer Lung cancer,	(107)
	Ivonescimab	Inhibiting PD-1 and VEGF-mediated angiogenesis	NSCLC Breast cancer	(108)
Cell therapy	CAR-T	Genetically engineered autologous T cells	B cell leukemia Lymphoma	(66)
	CAR-NK	Genetically engineered autologous NK cells	Etastatic pancreatic cancer”	(109)

The use of CIRT to trigger the release of tumor antigens and create an *in situ* vaccination effect constitutes a viable therapeutic strategy. Personalized mRNA vaccines (RNA-LPX), a novel class of therapeutics, are engineered to encode patient-specific tumor neoantigens derived from somatic mutations. This platform activates the host immune system to recognize and eliminate malignant cells (111) precisely. In a comparative study using colorectal cancer models, Nadja Salomon et al. evaluated the antitumor efficacy of combining CIRT with RNA-LPX vaccination versus X-ray irradiation with RNA-LPX. Results demonstrated CIRT’s superiority over X-ray in inducing tumor cell death and modulating antitumor immunity (112). These findings collectively indicate that combining CIRT with RNA-LPX vaccination represents a promising therapeutic strategy.

## 5 Perspectives and challenges

Immunotherapy is a relatively novel therapeutic approach, and the associated research remains comparatively limited in contrast to other treatment modalities. Due to insufficient patient enrollment, research on the efficacy of combined chemotherapy and CIRT had to be terminated prematurely (64, 113). For the same reason, clinical studies investigating the combination of immunotherapy and CIRT remain scarce. However, numerous studies have demonstrated that this combined approach can not only achieve tumor ablation and activate tumor-derived antigens to induce an anti-tumor vaccination effect, but may also benefit cancer patients through systemic immune activation. Furthermore, this method is not limited to malignant diseases; current research is exploring its potential applications in non-malignant conditions (114).

Currently, the most critical factors limiting the development of CIRT are the prohibitively high cost of the required technical equipment and the consequent cascade of challenges: high treatment expenses, a limited patient pool, difficulties in generating large-scale clinical data, and a lack of prospective studies. The advancement of CIRT in combination with immunotherapy is constrained not only by these intrinsic limitations of CIRT technology itself but also by complexities in the optimal timing, and dosing of the combined regimens. Furthermore, preclinical research on CIRT has not yet been widely adopted globally, and clinical studies remain largely confined to a limited number of specialized institutes. These issues represent critical challenges that must be urgently addressed to advance the field of CIRT. On the other hand, while extensive preclinical studies have demonstrated that the combination of CIRT and immunotherapy holds promise for clinical translation, the translation of such combined approaches into clinical practice still faces significant challenges due to species differences and the more complex physiological environment in humans.

In summary, extensive studies indicate that, compared to XRT, CIRT exhibit superior biological properties, including higher LET and enhanced immunogenicity. These characteristics significantly alter cellular proliferation and death pathways, ultimately amplifying immunogenic responses (115). However, optimizing combined CIRT and immunotherapy regimens requires careful consideration of multiple factors: (1) selection of optimal immune agents and radiation dose scheduling, (2) required damage thresholds and dose parameters, (3) lymphocyte status (116), and (4) standard tissue protection strategies (117). However, several critical challenges hinder widespread adoption: the inherent technical complexity of CIRT systems, substantial infrastructure costs, and elevated treatment expenses (118). These limitations result in small patient cohorts and a scarcity of large-scale Phase III clinical trials. Addressing these barriers remains imperative for advancing the field.

## 6 Summary

Overall, current evidence strongly indicates that CIRT possess significant immunological advantages and that their combination with immunotherapy is feasible. Nevertheless, studies on the radiation toxicity and immune-related side effects caused by this combined treatment remain insufficient, particularly in clinical research. This gap is largely due to the limitations of existing studies, including the high cost of CIRT and the relatively small number of patients receiving such treatments. Based on the above analysis, future research should focus more on investigating the mechanisms underlying the immunological characteristics of CIRT, translating their immunological advantages into clinical applications, and promoting the study of this combined treatment model. In summary, preliminary progress has been made in the research on combined CIRT and immunotherapy, and the current findings have laid a solid foundation for guiding future clinical cancer treatments. This will undoubtedly propel the combined therapy into a new phase of development.
